# Associated factors of overweight in adolescents from public schools in Northern Minas Gerais State, Brazil

**DOI:** 10.1590/0103-0582201432216213

**Published:** 2014-06

**Authors:** Lucinéia de Pinho, Ana Cristina de C. Botelho, Antônio Prates Caldeira

**Affiliations:** 1 Unimontes, Montes Claros, MG, Brasil

**Keywords:** adolescent, body mass index, nutrition assessment, obesity

## Abstract

**OBJECTIVE:**

In order to support plans and actions that combat the local increasing overweight and obesity prevalence in adolescents, the factors associated to weight excess in public school students from Montes Claros, MG, Southeast Brazil, were studied.

**METHODS:**

Cross-sectional study with a sample of adolescents from the public schools of the city. The nutritional status was evaluated and an inquiry was carried out in the schools to determine food consumption and practice of physical activities. Factors associated to weight excess were assessed by bivariate analysis followed by logistic regression.

**RESULTS:**

Weight excess prevalence was detected in 18.5% of the 535 adolescents evaluated. The factors associated to weight excess were: *per capita* income above half minimum wage (OR 1.99; 95%CI 1.01-3.93), candy consumption above two daily portions (OR 1.94; 95%CI 1.13-3.32) and absence of sport activity during leisure time (OR 2.54; 95%CI 1.15-5.59).

**CONCLUSIONS:**

The proportion of weight excess in adolescents from public schools is relevant and associated with socioeconomic condition of the family, bad eating habits and sedentary life.

## Abstract (es)

**OBJETIVO:**

A fin de subsidiar planes y acciones de combate local a la creciente prevalencia de sobrepeso y obesidad en adolescentes, se evaluaron los factores asociados al exceso de peso en estudiantes de la red pública de enseñanza en la ciudad de Montes Claros, Minas Gerais.

**MÉTODOS:**

Estudio transversal con muestra de adolescentes de la red pública de enseñanza municipal. Se evaluó el estado nutricional y, mediante un cuestionario en ambiente escolar, se determinó el consumo alimentar y la práctica de ejercicios físicos. Para identificar los factores asociados al exceso de peso, se realizaron análisis bivariadas seguidas de regresión logística, adoptándose el nivel de significancia de 5%.

**RESULTADOS:**

Se evaluaron 535 adolescentes, en los que la prevalencia de exceso de peso fue de 18,5%. Los factores asociados a la ocurrencia del exceso de peso fueron: ingreso per cápita superior a medio sueldo mínimo (OR 1,99; IC95% 1,01-3,93), consumo de dulces superior a dos porciones diarias (OR 1,94; IC95% 1,13-3,32) y ausencia de práctica de deportes en el tiempo de ocio (OR 2,54; IC95% 1,15-5,59).

**CONCLUSIONES:**

El exceso de peso tiene proporción relevante en adolescentes de escuelas públicas, asociándose a la condición socioeconómica de las familias, a los malos hábitos alimentares y al sedentarismo.

## Introduction

Adolescence extends from 10 to 19 years of age and involves complex somatic, psychological and social transformations^(^ 1 ^)^. It is a dynamic process and there is considerable intraindividual variation in body composition, which is influenced by factors such as inheritance, diet, physical activity, age and sex^(^ 1 ^)^.

The changes in body composition during adolescence are associated with metabolic changes which, in turn, may predict risk of emergence of chronic non-communicable disease in adulthood. One such factor is obesity, which is an emerging problem among Brazilian children and adolescents^(^ 2 ^)^. If the development of obesity is to be avoided and appropriate interventions are to be planned and implemented, it is of fundamental importance to monitor the nutritional status of adolescents^(^ 3 ^)^. 

Body mass index (BMI), calculated by dividing weight by the square of height, has become one of the most widely-used indicators for assessment of the nutritional status of adolescents. The measure is universally applicable due to its low cost, simplicity and high reproducibility and it effectively differentiates excess body fat in adolescents^(^ 4 ^,^ 5 ^)^. Body mass index is recommended by the World Health Organization^(^ 6 ^)^ and has been utilized in epidemiological studies^(^ 2 ^,^ 7 ^,^ 8 ^)^ and the elevated BMI values that characterize overweight and obesity are associated with numerous organic complications^(^ 9 ^)^.

Simply measuring BMI, however, is not in itself enough to provide a foundation for actions to prevent and combat obesity. It is also necessary to identify the factors that are determinant of, or associated with, excess weight, especially those linked to the diet and the practice of physical activity. It is therefore necessary to profile the study population's nutritional and physical activity habits in order to widen the nutritional assessment. It is known that nutritional education combined with physical activity is capable of reducing the BMI of adolescents^(^ 10 ^)^.

Studies of obesity are conducted all over the world, but actions to combat overweight must be of a regional nature, since dietary and behavioral habits are strongly affected by socioeconomic and cultural aspects of communities. In view of the above, the objective of this study was to identify factors associated with excess weight in adolescents from public schools in the North of the state of Minas Gerais, Brazil.

## Method

This cross-sectional and analytical study was conduced during the second 6 months of 2011 with adolescents of both sexes aged 11 to 17 years and enrolled at public schools run by the municipal education authority in the urban zone of Montes Claros, MG, Brazil.

A sample size of 474 individuals was estimated using Epi-Info version 3.5.2, based on the total number of pupils enrolled from year six to year nine at schools run by the public education system in the municipal district's urban zone (the rural areas were excluded for logistical motives and because they account for less than 3% of the local public education system's students). In addition to the entirety of the student roll, the sample size calculation was also based on a 20% prevalence of obesity (according to data from surveys conducted by the Brazilian Institute of Geography and Statistics [IGBE - Instituto Brasileira de Geografia e Estatística] with Brazilian adolescents)^(^ 11 ^)^ a 95% confidence level, a sampling error of 5% and a correction factor for the sampling design (*deff*) of 2.

The sample was selected at random using two-stage cluster sampling. In the first stage, schools were selected using size-proportional probabilities. In the second stage, classes were selected from these schools by simple random sampling and then all students in the class so selected were interviewed^(^ 8 ^)^.

Sociodemographic data on the participants were collected (sex, age, socioeconomic status and educational level of parents). Parents provided information on age by age group and socioeconomic status on a form. Participants were classified by per capita income in terms of the minimum monthly wage (based on the income received by the family during the month prior to the interview) as <1/2 minimum wage or ≥1/2 minimum wage. 

Habitual food intakes were assessed using a Food Frequency Questionnaire for Adolescents (FFQA). This is a semiquantitative instrument offering seven intake options for 94 foods, as follows: never; less than once a month; from one to three times per month; once a week; two to four times per week; once a day; two or more times per day^(^ 12 ^)^. The adolescents responded to this questionnaire themselves.

The data collected using the FFQA were input to a spreadsheet for analysis of the nutritional value of individual diets. The intake frequencies for food items were converted into daily values. The software program Diet Pró^(r)^ was used to conduct nutritional calculations for all of the foods eaten. From this, the number of daily portions of fruit, vegetables, sugary foods and fats each participant consumed were calculated. Adolescents who reported eating a minimum of three daily portions of fruit and vegetables and a maximum of two portions of sugary foods were defined as exposed to an appropriate pattern of consumption of these foods.

In order to preserve the quality of data, extreme datasets were excluded from the analysis, i.e. questionnaires that suggested individuals had an energy intake of less than 500 calories (5 items on the FFQA) or greater than 7000 calories (51 items on the FFQA).

The adolescents' practice of physical activity was analyzed using a questionnaire proposed by Barros et al^(^ 13 ^)^ which analyzes a typical day's physical activities and nutrition. For answers to questions about means of transport used for daily displacement from home to school, walking and cycling were defined as active displacement while transport by car, motorbike or bus were defined as passive displacement. 

Anthropometric weight and height measurements were taken. Adolescents were weighed wearing light clothing and unshod on a portable class III electronic balance (Marte^(r)^ LC200-PS), with a maximum capacity of 199.95kg, minimum capacity of 1kg and precision of 50g.

Height was measured using a vertical stadiometer (Altura Exata^(r))^ with a bilateral numerical scale from 35-213cm with 0.1cm divisions. For this measurement, adolescents remained unshod and were positioned with feet together, heels against the wall, in an erect position, with gaze fixed on the horizon and with no flexion or extension of the head. The stadiometer's horizontal bar was then lowered until it rested on top of the subject's head, at which point their height was read off in centimeters.

Weight and height were measured in duplicate and the means used to calculate BMI for age (Z scores) in order to assess nutritional status. The World Health Organization reference values for children and adolescents from 5 to 19 years were used^(^ 14 ^)^. For the purposes of analysis, adolescents were defined as healthy weight (underweight and normal weight) or excess weight (overweight and obesity) and the second of these was considered the outcome variable. 

Before conducting the research proper, a pilot study was conducted with 26 adolescents of both sexes in order to perfect instruments and methodological procedures. Once this phase was complete, data collection was conducted during the second half of 2011 in a specially chosen location at each school, during lesson time, by a team of investigators who had been trained and calibrated in advance (interexaminer Kappa: 0.60; intraexaminer Kappa: 0.74).

Statistical treatment of data was conducted using the Statistical Package for the Social Sciences (SPSS), version 15.0. Absolute and relative frequencies were used to describe sociodemographic characteristics, dietary intake and physical activity by nutritional status (healthy weight versus excess weight).

Statistical analysis of associations between independent factors and the dependent variable "excess weight" was conducted using binary, univariate and multivariate logistic regression models. The multivariate analysis was used to test those variables that had a descriptive level of less than 20% and those that, according to theoretical references, may explain behavior. The final model contains those variables with statistical significance to 5%. 

This study was conducted in compliance with ethical principles. Initially, adolescents were provided with explanations about the study and those who agreed to take part were given a consent form to be signed by parents or guardians. The study was approved by the Research Ethics Committee at the Universidade Estadual de Montes Claros, process nº 3016/2011. 

## Results

The study population comprised 535 adolescents aged 11-17 years, 68.0% of whom were female (n=364). The prevalence of excess weight was 18.5% (95%CI 15.4-22.2).

Analysis of sociodemographic characteristics revealed that excess weight was associated with income (*p*=0.026). Adolescents with per capita incomes greater than half of the minimum wage were 2.11 times more likely to have excess weight (Table 1). 

**Table 1:**
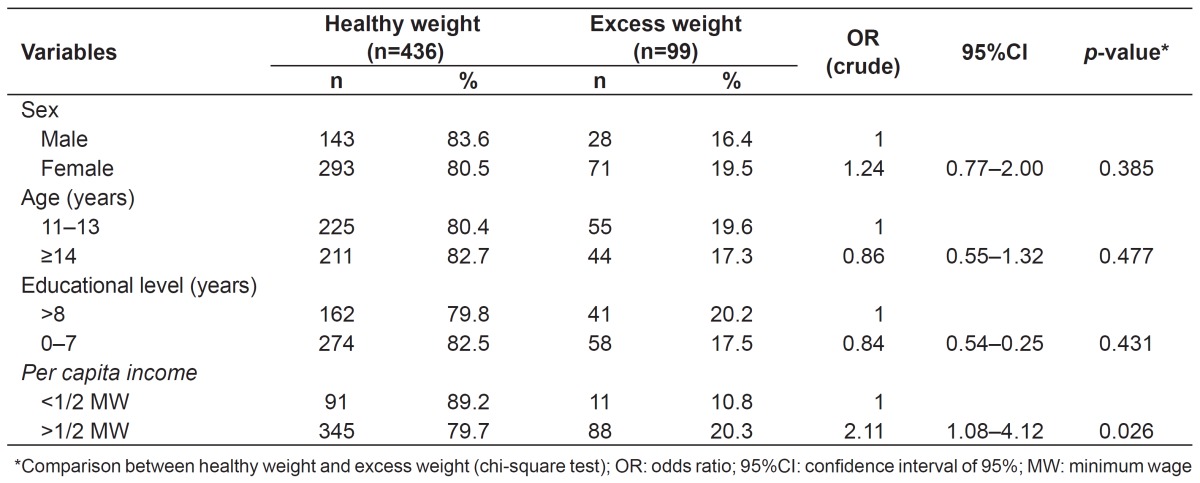
Comparison of adolescents' sociodemographic variables, stratified by nutritional status

Table 2 shows the results of the bivariate analyses conducted to test for associations between excess weight and dietary intake. High frequency of consumption of sugary foods was significantly associated with excess weight in these adolescents (*p*=0.017). It was observed that more than half of the adolescents were not consuming the minimum recommendation of three portions of fruit and vegetables per day.

**Table 2:**
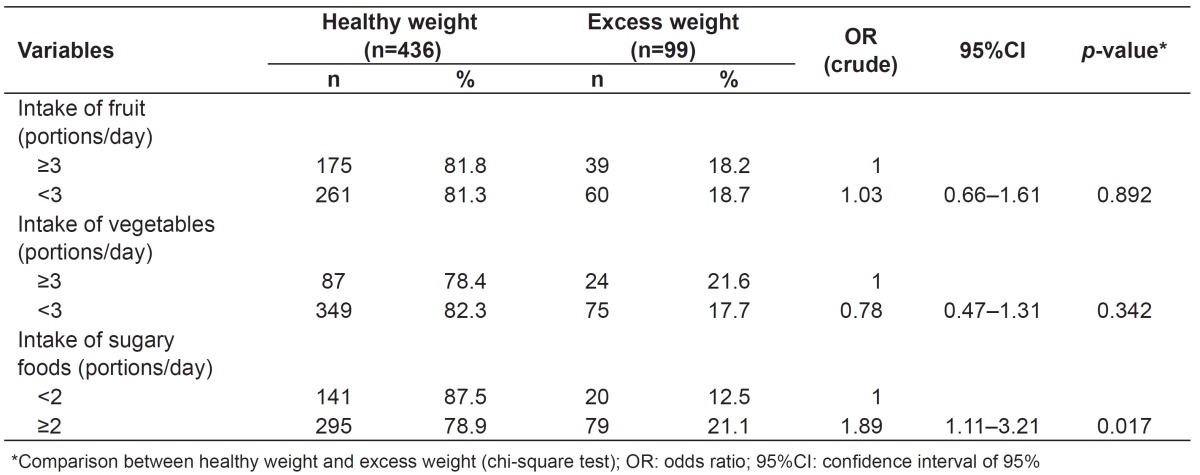
Comparison of dietary intake variables for adolescents, stratified by nutritional status

With regard to the practice of physical activity among these adolescents (Table 3), it was observed that only the variable "sporting activities during leisure time" had a statistically significant negative association (*p*=0.009) with excess weight. In other words, there was a higher prevalence of excess weight among those who reported not engaging in sports. The results also show that approximately 80% of these adolescents used active methods of displacement to school, spending less than 15 minutes to make the journey. Almost half of the adolescents engaged in sedentary activities (TV and/or computer) and did not perform domestic chores during leisure time. Around 80% of adolescents did not engage in recreational activities. 

**Table 3:**
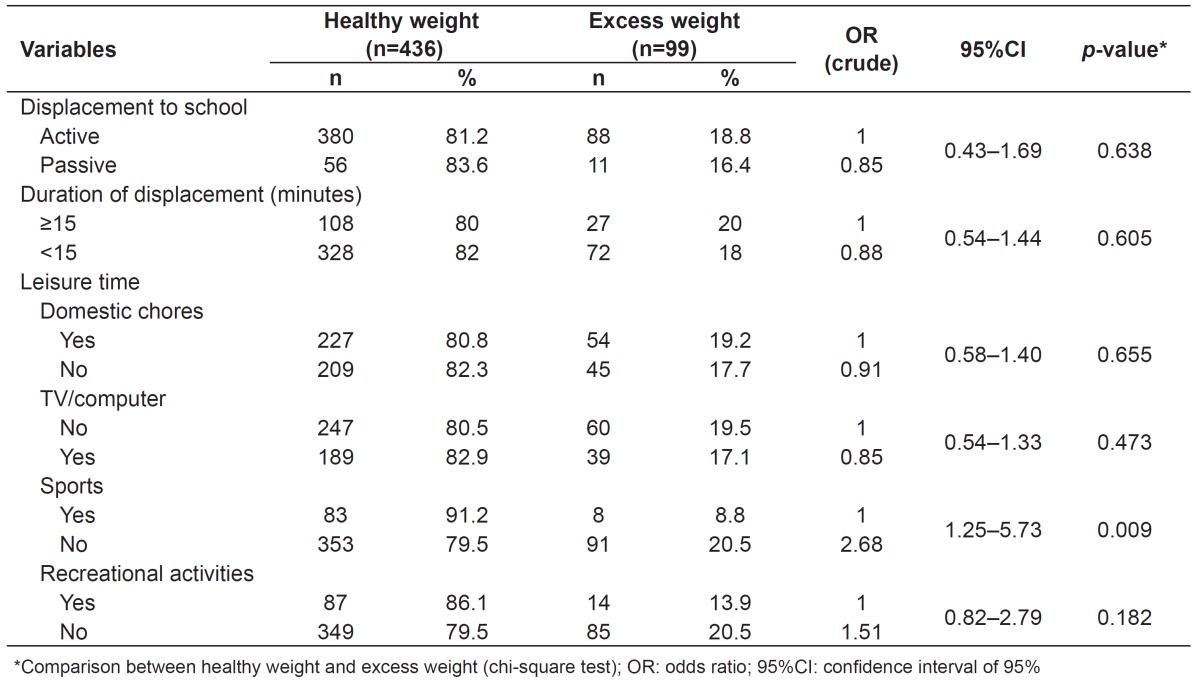
Comparison of physical activity variables for adolescents, stratified by nutritional status

The multiple analysis (Table 4) identified per capita income greater than half of the minimum wage, high intake of sugary foods and an absence of sporting activities as factors that promote excess weight among these adolescents (*p*=0.05). 

**Table 4:**
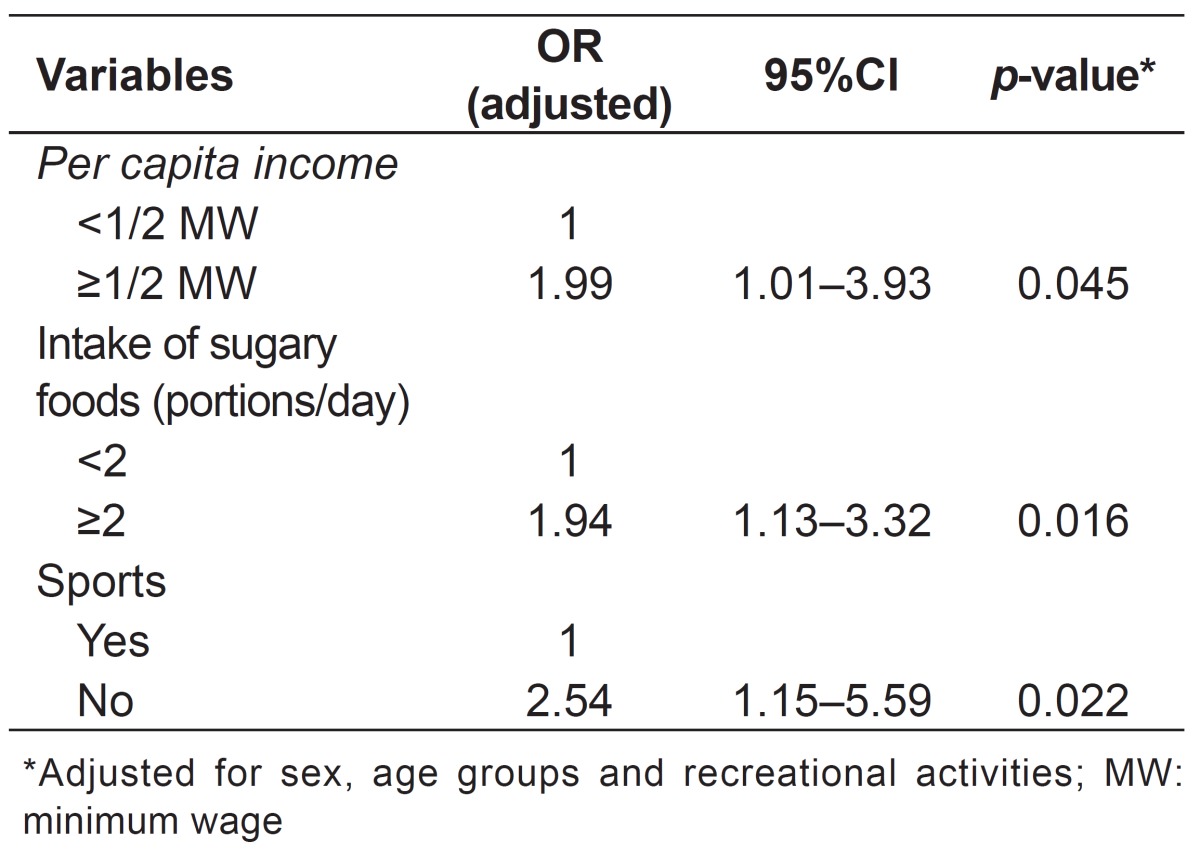
Multiple logistic regression analysis of factors associated with excess weight in adolescents from Montes Claros, MG, Brazil

## Discussion

While it is clear that there is an elevated frequency of overweight and obesity among Brazilian adolescents, understanding of the principal modifiable risk factors responsible for current trends is still limited^(^ 1 ^)^. In view of this, this study was conducted to investigate which variables are determinants of the nutritional status of adolescents enrolled in the public education system of Montes Claros, Brazil. The factors identified as associated were socioeconomic status of adolescents' families, unhealthy dietary habits and inactivity. Considering that dietary and lifestyle habits that are consolidated in adolescence are established by adulthood, it is essential to monitor these behaviors in order to promote the health of this group.

Of the candidate variables chosen to explore associations between excess weight and sociodemographic characteristics, income exhibited a significant association: adolescents with higher incomes had higher prevalence rates of excess weight. In developing countries, aspects linked to socioeconomic characteristics, and particularly income, are determinants of obesity in adolescents^(^ 15 ^,^ 16 ^)^. According to data from the Family Budgets Survey (*Pesquisa de Orçamentos Familiares*) conducted in 2008-2009, income is directly related to excess weight, i.e. there is a higher prevalence of excess weight among adolescents with higher income than among those with lower income^(^ 17 ^)^. This association has also been reported in other regions of Brazil, as shown by a cross-sectional population study of children and adolescents in Pernambuco, which found that higher family incomes were among the determinants of excess body weight^(^ 18 ^)^. Therefore, socioeconomic status can be considered an important determinant of the prevalence of excess weight, interfering with the ability to acquire food.

Unhealthy dietary behavior and inactivity are generally identified as factors associated with obesity. With regard to dietary behavior, the results of the present study identified an association between excess weight and intakes of sugary foods greater than two portions per day, with a prevalence ratio of 1.89 times greater likelihood of the person having excess weight when compared with those who have lower intakes of sugary foods. Adolescents consume sugars in excess, primarily because of consumption of sugar-based drinks, characterizing an inappropriate dietary profile that confers risk to health^(^ 19 ^)^. Petribú et al^(20) ^conducted a study to investigate prevalence of overweight and obesity and identify factors associated with them in Secondary Education students at state-run public schools in the municipal district of Caruaru, PE, Brazil, reporting that those who consumed sugary foods with a frequency greater than or equal to four times per week had a 3.98 greater chance of being obese than those who reported consuming sugary foods three times per week or less^(^ 20 ^)^. They also found that intakes of fruit and vegetables were not associated with obesity. The present study confirms their findings. 

More than 50% of the adolescents assessed were not consuming the minimum recommendation of three portions of fruit and vegetables a day. This result was expected and has been reported by other studies conducted in Brazil^(^ 21 ^,^ 22 ^)^. The National Adolescent School-based Health Survey (PeNSE) investigated the characteristics of dietary intakes and behaviors of Brazilian adolescents and found that just 30% had the recommended intakes of fruit and vegetables^(^ 23 ^)^. In general, intakes of these food groups are critical for adolescents, irrespective of nutritional status. Intersectorial interventions to encourage consumption of fruit and vegetables and the adoption of a healthy lifestyle are promising options for the fight against obesity^(^ 24 ^)^, especially considering the role played by different types of carbohydrates and regulation of appetite, by body weight and by body composition^(^ 19 ^)^.

Recent studies into the factors associated with overweight and obesity have tended to consider exposure to sedentary behaviors in leisure activities, in addition to physical activity^(^ 2 ^,^ 20 ^,^ 25 ^-^ 27 ^)^. In the present study, it was observed that not practicing sport was associated with excess weight in adolescents. Almost half of these adolescents engaged in activities such as watching television or using computers during leisure time, but this variable was not related to excess weight. Although other studies have also failed to find an association between practicing activities demanding low energy expenditure, such as using TV/computer, and obesity, it is possible that investigation of data specifically on "screen time" would detect a relationship between these variables^ (2,28)^. In general, the adolescents described here were insufficiently active during their leisure time, as shown by the low level of participation in recreational activities. This profile has also been observed in other studies with Brazilian adolescents^(^ 25 ^,^ 27 ^,^ 29 ^)^. 

Dietary behavior and physical activity are generally identified as factors associated with obesity. However, epidemiological studies have demonstrated that this inter-relationship has not yet been sufficiently elucidated, since these variables are difficult to measure, especially in adolescents^(^ 1 ^)^.

One limitation of this study was its cross-sectional design, which does not provide evidence for making statements on cause and effect. The findings should therefore be treated with caution until a longitudinal investigation of modifiable risk factors for obesity is conducted with young Brazilians. Although studies of a cross-sectional nature do not allow for inferences of causality, they are important for generating hypotheses and for guiding planning of prospective studies, which, in turn, can establish clearer relationships between factors related to lifestyle and nutritional status in adolescents. Another limitation is that puberty stages were not assessed, which is a variable to be investigating future research. 

Despite these limitations, this study is the first of its type in the region and represents the universe of municipal schools in Montes Claros. The results of this investigation show that, of the variables analyzed, income, intake of sugary foods and engagement in sporting activities were associated with excess weight among adolescents. The results can be used to provide a basis for political and educational measures, which are useful for combating and preventing the worsening problem of high prevalence rates of overweight and obesity among adolescents.
