# Supplementary material for: Associated factors of overweight in adolescents from public schools in Northern Minas Gerais State, Brazil
Source: Rev Paul Pediatr. 2014 Jun;32(2):237–43. doi: 10.1590/0103-0582201432216213 (PMC4183019; doi:10.1590/0103-0582201432216213)
Supplement: Translation

# Translation: Fatores associados ao excesso de peso em adolescentes de escolas públicas no norte de Minas Gerais

**Authors:** Lucinéia de Pinho, Ana Cristina de C. Botelho, Antônio Prates Caldeira

**Affiliations:** 1 Unimontes, Montes Claros, MG, Brasil

**Keywords:** adolescente, índice de massa corporal, avaliação nutricional, obesidade

## Abstract

**OBJETIVO:**

A fim de subsidiar planos e ações de combate local à crescente prevalência de sobrepeso e obesidade em adolescentes, avaliaram-se os fatores associados ao excesso de peso em estudantes da rede pública de ensino na cidade de Montes Claros, MG.

**MÉTODOS:**

Estudo transversal com amostra de adolescentes da rede pública de ensino municipal. Avaliou-se o estado nutricional e, por meio de inquérito em ambiente escolar, determinou-se o consumo alimentar e a prática de exercícios físicos. Para identificar os fatores associados ao excesso de peso, realizaram-se análises bivariadas seguidas de regressão logística.

**RESULTADOS:**

Avaliaram-se 535 adolescentes, nos quais a prevalência de excesso de peso foi de 18,5%. Os fatores associados à ocorrência do excesso de peso foram: renda *per capita* superior a meio salário mínimo (OR 1,99; IC95% 1,01-3,93), consumo de doces superior a duas porções diárias (OR 1,94; IC95% 1,13-3,32) e ausência de prática de esporte no tempo de lazer (OR 2,54; IC95% 1,15-5,59).

**CONCLUSÕES:**

O excesso de peso tem proporção relevante em adolescentes de escolas públicas, associando-se à condição socioeconômica das famílias, aos maus hábitos alimentares e ao sedentarismo.

## Introdução

A adolescência se estende dos dez aos 19 anos de idade e compreende complexas transformações somáticas, psicológicas e sociais^(^ ^1^ ^)^. Nesse processo dinâmico, há expressiva variação da composição corporal entre indivíduos, que pode ser influenciada por fatores como hereditariedade, dieta, atividade física, idade e sexo^(^ ^1^ ^)^.

Modificações na composição corporal de adolescentes associam-se a alterações metabólicas que, por sua vez, podem predizer o risco de ocorrência de algumas morbidades crônicas não transmissíveis na vida adulta. É o caso da obesidade, problema emergente em crianças e adolescentes brasileiros^(^ ^2^ ^)^. Para evitar seu desenvolvimento, bem como planejar e implementar medidas de intervenção adequadas, é fundamental que se monitore o estado nutricional dos adolescentes^(^ ^3^ ^)^. 

O índice de massa corpórea (IMC), expresso pela relação entre o peso e o quadrado da altura, tem sido um dos indicadores mais utilizados para avaliar o estado nutricional de adolescentes. Acessível e universalmente aplicável devido ao baixo custo, à simplicidade e à alta reprodutibilidade, o IMC é eficiente ao discriminar o excesso de gordura corporal em adolescentes^(^ ^4^ ^,^ ^5^ ^)^. Recomendado pela Organização Mundial da Saúde^(^ ^6^ ^)^, esse indicador tem sido aplicado em estudos epidemiológicos^(^ ^2^ ^,^ ^7^ ^,^ ^8^ ^)^, sendo que valores elevados de IMC caracterizam sobrepeso e obesidade e associam-se a várias complicações orgânicas^(^ ^9^ ^)^.

A simples aferição do IMC, entretanto, não é suficiente para subsidiar ações de prevenção e combate à obesidade. É preciso também conhecer os fatores determinantes ou associados ao excesso de peso, especialmente aqueles relacionados à dieta e à realização de atividades físicas. Nesse sentido, para ampliar a avaliação nutricional, é importante identificar os hábitos nutricionais da população estudada e sua prática de atividades físicas. Sabe-se que a educação nutricional combinada à atividade física é capaz de reduzir o IMC de adolescentes^(^ ^10^ ^)^.

Estudos sobre obesidade são desenvolvidos em todas as partes do mundo, mas as ações de combate devem ter caráter regional, visto que hábitos alimentares e comportamentais são fortemente afetados por aspectos socioeconômicos e culturais das comunidades. Nesse contexto, o objetivo deste estudo foi identificar os fatores associados ao excesso de peso em adolescentes de escolas públicas no norte do Estado de Minas Gerais, Brasil.

## Método

Estudo analítico e transversal realizado no segundo semestre de 2011 com adolescentes de ambos os gêneros, de 11 a 17 anos, matriculados na rede pública de ensino municipal na zona urbana de Montes Claros, MG.

Calculou-se o tamanho amostral de 474 indivíduos com o *software* Epi-Info versão 3.5.2 e com base no número total de alunos de ensino fundamental matriculados do sexto ao nono ano de escolas da rede pública da zona urbana do município (excluiu-se a zona rural por questões de logística e por atender a menos de 3% dos estudantes da rede pública local). Além do universo de alunos matriculados, considerou-se, para o cálculo amostral, prevalência de obesidade de 20% (segundo dados de estudos do Instituto Brasileiro de Geografia e Estatística com adolescentes brasileiros)^(^ ^11^ ^)^, nível de confiança de 95%, erro amostral de 5% e fator de correção para o desenho amostral (*deff*) igual a 2.

Escolheu-se a amostra aleatoriamente, conforme a técnica de amostragem por conglomerados em dois estágios. No primeiro estágio, selecionaram-se as escolas por meio de método de seleção com probabilidades proporcionais ao tamanho. No segundo, selecionaram-se as turmas por amostragem aleatória simples, entrevistando-se todos os alunos das turmas selecionadas^(^ ^8^ ^)^. 

Coletaram-se informações sociodemográficas (gênero, idade, nível socioeconômico e escolaridade dos pais) dos participantes. A idade considerada por faixa etária e o nível socioeconômico foram declarados pelos pais em um formulário. Classificaram-se os sujeitos conforme a renda *per capita *em salários mínimos (obtidos pela família no mês anterior à entrevista), isto é, em <1/2 salário mínimo ou ≥1/2 salário mínimo. 

Avaliou-se o consumo habitual de alimentos por meio do Questionário de Frequência Alimentar para Adolescentes (QFAA). Trata-se de um instrumento semiquantitativo que oferece sete opções de consumo para 94 alimentos: nunca; menos de uma vez ao mês; de uma a três vezes no mês; uma vez por semana; duas a quatro vezes por semana; uma vez ao dia; duas ou mais vezes ao dia^(^ ^12^ ^)^. O questionário foi respondido pelos próprios adolescentes.

Organizaram-se os dados do QFAA em planilha para analisar o valor nutricional das dietas individuais. As frequências de consumo dos diferentes itens alimentares foram transformadas para valores diários. Utilizando-se o *software* Diet Pró^(r)^, realizaram-se os cálculos nutricionais para todos os alimentos ingeridos. Assim, considerou-se, para cada indivíduo, o número de porções diárias de frutas, hortaliças, doces e gorduras consumidas. Os adolescentes que relataram consumo diário de frutas e verduras de, no mínimo, três porções diárias e de, no máximo, duas porções de doces (açúcares) foram classificados como expostos ao padrão adequado de consumo desses alimentos.

Para manter a qualidade dos dados, excluíram-se da pesquisa as situações extremas, ou seja, as informações referentes aos indivíduos cuja ingestão energética apresentava-se inferior a 500 calorias (cinco itens do QFAA) ou superior a 7 mil calorias (51 itens do QFAA).

Analisou-se a prática de atividade física nos adolescentes com base no questionário proposto por Barros *et al**^(^* ^13^ ^)^, que avalia um dia típico de atividades físicas e alimentação (DAFA). Na análise das respostas sobre o meio de transporte utilizado para se deslocar de casa até a escola, considerou-se a caminhada e o ciclismo como deslocamento ativo e o uso de carro, moto e ônibus como deslocamento passivo. 

Avaliaram-se as medidas antropométricas de peso e estatura. Para aferir o peso corporal, solicitou-se que os adolescentes vestissem roupas leves e permanecessem descalços, sendo então posicionados sobre uma balança eletrônica portátil Marte^(r)^ classe III, modelo LC200-PS, com capacidade para 199,95kg, capacidade mínima de 1kg e precisão de 50g.

Para a medida da altura, utilizou-se estadiômetro vertical Altura Exata^(r)^ com escala numérica bilateral de 35-213cm e resolução de 0,1cm. Nessa medição, os jovens mantinham-se descalços, com os pés juntos, calcanhares encostados na parede, em postura ereta, com olhar fixo no horizonte, sem flexão ou extensão da cabeça. Em seguida, posicionava-se a barra horizontal do estadiômetro até mantê-la apoiada sobre a cabeça do adolescente, sendo então efetuada a leitura da altura em centímetros.

Mediram-se o peso e a altura em duplicata e o valor médio obtido para cada participante foi usado para calcular o IMC para idade (em escores Z) a fim de avaliar o estado nutricional. Adotou-se como referência a proposta da Organização Mundial da Saúde para crianças e adolescentes de cinco a 19 anos^(^ ^14^ ^)^. Para fins de análise, categorizaram-se os adolescentes em eutróficos (baixo peso e eutrófico) e com excesso de peso (sobrepeso e obeso), sendo o último grupo considerado variável desfecho. 

Antes de iniciar a pesquisa, realizou-se um estudo-piloto com 26 adolescentes de ambos os gêneros a fim de aperfeiçoar os instrumentos e os procedimentos metodológicos. Completada essa fase, a coleta de dados - efetuada no segundo semestre de 2011 - foi conduzida em espaço reservado de cada escola durante a jornada de aula, por equipe de pesquisadores previamente treinados e calibrados (Kappa interexaminadores: 0,60; Kappa intraexaminadores: 0,74).

Realizou-se o tratamento estatístico das informações com o programa *Statistical Package for the Social Sciences* (SPSS), versão 15.0. Para descrever as características sociodemográficas, o consumo alimentar e a atividade física da amostra segundo o estado nutricional (eutrófico e com excesso de peso), utilizaram-se frequências absolutas e relativas.

A análise estatística da associação entre os fatores independentes e a variável dependente "excesso de peso" foi feita por modelos de regressão logística binária, univariados e múltiplos. Para a análise múltipla, testaram-se as variáveis que apresentaram nível descritivo inferior a 20% e aquelas que poderiam explicar algum comportamento, segundo referenciais teóricos. Permaneceram no modelo final as variáveis que apresentaram significância estatística de até 5%. 

Conduziu-se o estudo dentro dos preceitos éticos. Inicialmente, os adolescentes foram informados sobre o estudo e aqueles que concordaram em participar receberam um termo de consentimento para ser assinado pelos pais ou responsáveis. O estudo foi aprovado pelo Comitê de Ética em Pesquisa da Universidade Estadual de Montes Claros. 

## Resultados

A população estudada foi composta por 535 adolescentes de 11-17 anos de idade, sendo 68,0% (n=364) do gênero feminino. A prevalência de excesso de peso foi de 18,5% (IC95% 15,4-22,2%).

Na análise das características sociodemográficas, o excesso de peso foi associado à renda (*p*=0,026). Adolescentes provenientes de famílias com renda *per capita* superior a meio salário mínimo apresentaram chance 2,11 vezes maior de estarem com excesso de peso (Tabela 1). 

**Tabela 1:** Comparação das variáveis sociodemográficas de adolescentes estratificados conforme o estado nutricional

A Tabela 2 mostra os resultados da análise bivariada para testar associações entre excesso de peso e consumo alimentar. A alta frequência de consumo de doces foi significativamente associada ao excesso de peso em adolescentes (*p*=0,017). Observou-se que mais da metade dos adolescentes não consumia a recomendação mínima de três porções de frutas e verduras ao dia.

**Tabela 2:** Comparação do consumo alimentar de adolescentes estratificados conforme o estado nutricional

Quanto à prática de atividade física entre os adolescentes (Tabela 3), observou-se que apenas a variável "atividade esportiva no tempo de lazer" apresentou associação significante (*p*=0,009) negativa com excesso de peso, isto é, a prevalência de excesso de peso foi maior naqueles que relataram não praticar esportes. Os resultados demonstraram que aproximadamente 80% dos adolescentes deslocavam-se ativamente à escola, com distância percorrida em tempo menor que 15 minutos. Quase metade dos adolescentes participava de atividades sedentárias (TV e/ou computador) e não realizava tarefas domésticas no tempo de lazer. Cerca de 80% dos adolescentes não praticavam atividades recreativas. 

**Tabela 3:** Comparação da atividade física de adolescentes estratificados conforme o estado nutricional

Na análise múltipla (Tabela 4), a renda *per capita* superior a meio salário mínimo, o alto consumo de doces e a ausência de atividades esportivas permaneceram como fator promotor do excesso de peso nos adolescentes (*p*<0,05). 

**Tabela 4:** Análise de regressão logística múltipla para fatores associados ao excesso de peso em adolescentes de Montes Claros, MG

## Discussão

Embora seja claro que os adolescentes brasileiros apresentem elevada frequência de sobrepeso e obesidade, a compreensão dos principais fatores de risco modificáveis responsáveis pelas tendências atuais ainda é limitada^(^ ^1^ ^)^. Nesse sentido, o presente estudo investigou as variáveis determinantes do estado nutricional de adolescentes da rede de ensino de Montes Claros, detectando que os fatores associados são a condição socioeconômica das famílias, os hábitos alimentares inadequados e o sedentarismo. Considerando-se que os hábitos alimentares e de estilo de vida consolidados na adolescência são estabelecidos na vida adulta, monitorar esse comportamento é essencial para promover a saúde desse grupo.

Dentre as variáveis propostas no estudo para explorar a associação de excesso de peso com as características sociodemográficas, a renda apresentou associação significativa: os adolescentes com maior renda apresentaram maiores prevalências de excesso de peso. Em países em desenvolvimento, questões ligadas às características socioeconômicas, em especial a renda, são determinantes da obesidade em adolescentes^(^ ^15^ ^,^ ^16^ ^)^. Conforme dados da Pesquisa de Orçamentos Familiares (POF) realizada em 2008-2009, a renda é diretamente vinculada ao excesso de peso, ou seja, existe maior prevalência de excesso de peso em adolescentes de maior renda do que nos de menor renda^(^ ^17^ ^)^. Essa associação é relatada em outras regiões brasileiras, como mostra um estudo transversal e de base populacional com crianças e adolescentes de Pernambuco, o qual evidenciou que maiores rendas familiares estiveram entre os determinantes do excesso de peso ponderal^(^ ^18^ ^)^. Dessa forma, pode-se considerar o nível socioeconômico como um importante determinante da prevalência de excesso de peso, interferido na possibilidade de aquisição de alimentos.

O comportamento alimentar inadequado e o sedentarismo são geralmente apontados como fatores associados à obesidade. Quanto ao comportamento alimentar, a presente pesquisa evidenciou associação entre o consumo de doces superior a duas porções diárias e excesso de peso, com razão de prevalência de 1,89 vezes a chance de o indivíduo desenvolver excesso de peso quando comparado àqueles com consumo inferior a essa quantidade. Os açúcares são consumidos em excesso por adolescentes, principalmente devido ao uso de bebidas à base de açúcar na dieta, caracterizando um padrão alimentar inadequado e de risco à saúde^(^ ^19^ ^)^. Em estudo para investigar a prevalência e os fatores associados ao sobrepeso e à obesidade em estudantes do ensino médio da rede pública estadual do município de Caruaru, PE, Aller *et al**^(^* ^19^ ^)^ relataram que estudantes que consumiam doces com uma frequência maior ou igual a quatro vezes por semana tiveram chance 3,98 vezes maior de obesidade do que os que relataram consumo de doce inferior ou igual a três vezes por semana^(^ ^20^ ^)^. Verificaram ainda que o consumo de frutas e verduras não se associou à obesidade. O presente estudo confirma esses dados. 

Entre os adolescentes avaliados, mais de 50% não consumiram a recomendação mínima de três porções de frutas e verduras ao dia. Esse resultado já era esperado e foi relatado em outros estudos nacionais^(^ ^21^ ^,^ ^22^ ^)^. A Pesquisa Nacional de Saúde do Escolar (PeNSE) avaliou as características de consumo e comportamento alimentar de adolescentes brasileiros e verificou que apenas 30% consumiam frutas e verduras em quantidades recomendadas^(^ ^23^ ^)^. No geral, o consumo desses grupos de alimentos são críticos em adolescentes, independentemente do estado nutricional. No combate à obesidade, ações intersetoriais de incentivo ao consumo de frutas e verduras e a adoção de estilo de vida saudável são promissoras^(^ ^24^ ^)^, especialmente considerando-se o papel dos diferentes tipos de carboidratos e a regulação do apetite, do peso e da composição corporal^(^ ^19^ ^)^.

Estudos atuais sobre fatores associados a sobrepeso e obesidade tendem a considerar, além das práticas de atividade física, a exposição a comportamentos sedentários nas práticas de lazer^(^ ^2^ ^,^ ^20^ ^,^ ^25^ ^-^ ^27^ ^)^. No presente estudo, observou-se que a ausência de prática de esportes em adolescentes associou-se ao excesso de peso. Em seu tempo de lazer, quase metade dos adolescentes participava de atividades como assistir televisão ou fazer atividades no computador, mas esse dado não se relacionou ao excesso de peso. Embora outros estudos relatem a falta de associação entre a prática de atividades e a baixa demanda energética, como o uso de TV/computador e obesidade, é possível que a investigação de dados específicos sobre o "tempo de tela" detecte alguma relação entre tais variáveis^(^ ^2^ ^,^ ^28^ ^)^. De modo geral, os adolescentes eram insuficientemente ativos no tempo de lazer, como mostrado pela baixa participação em atividade de recreação. Observou-se esse perfil em outros estudos com adolescentes brasileiros^(^ ^25^ ^,^ ^27^ ^,^ ^29^ ^)^. 

O comportamento alimentar e a prática de atividade física são geralmente apontados como fatores associados à ocorrência de obesidade. No entanto, estudos epidemiológicos demonstraram que essa inter-relação não está suficientemente esclarecida, pois essas variáveis são difíceis de mensurar, especialmente em adolescentes^(^ ^1^ ^)^.

Uma limitação do estudo é o desenho transversal, que impede declarações de causa e efeito. Dessa forma, os achados devem ser tratados com cautela até que uma avaliação longitudinal de fatores de risco modificáveis para obesidade seja realizada em jovens brasileiros. Embora estudos de natureza transversal não permitam inferir causalidade, eles são importantes para gerar hipóteses e direcionar o planejamento de estudos prospectivos, que, por sua vez, podem estabelecer relações mais claras entre os fatores relacionados ao estilo de vida e o estado nutricional em adolescentes. Outra limitação é que não se avaliou o estágio puberal, sendo uma variável a ser explorada em pesquisas futuras. 

Apesar dessas limitações, o presente estudo é inédito na região e representa o universo das escolas municipais de Montes Claros. Os resultados obtidos nesta investigação revelam que, das variáveis analisadas, a renda, o consumo de doces e as atividades esportivas associaram-se ao excesso de peso dos adolescentes. Seus resultados podem ser utilizados para subsidiar medidas políticas e pedagógicas, úteis para enfrentar e evitar o agravamento do problema da alta prevalência de sobrepeso e obesidade em adolescentes.
